# The Time Course of Updating in Running Span

**DOI:** 10.1037/xlm0000800

**Published:** 2019-12-19

**Authors:** Shraddha Kaur, Dennis G. Norris, Susan E. Gathercole

**Affiliations:** 1 Medical Research Council Cognition and Brain Sciences Unit, University of Cambridge

**Keywords:** running span, working memory updating, cognitive demand, working memory

## Abstract

Running span can be performed by either passively listening to memory items or actively updating the target set. Previous research suggests that the active updating process is demanding and time consuming and is favored at slow rates of presentation while the passive strategy is employed at fast rates. Two experiments examined the time course of recruitment of resources during task performance and its sensitivity to presentation rate. In Experiment 1, participants performed 1 of 3 serial recall tasks: running span, simple span, and modified span. The tasks were completed at the same time as a choice reaction time (RT; CRT) task and the RTs were used to index the resource demands of the memory task. Running span generated higher RT costs than simple span. The costs were present only for positions at and beyond the point in the sequence when the target memory set was changed, indicating a shift to a more cognitively demanding mode of updating. At these positions there was a generalized increase in RT costs that peaked 1,000 ms following item presentation. In Experiment 2 the resource demands of running span varied with presentation rate and a peak demand at 1,000 ms was again evident, but only with a slow presentation rate. In conjunction with strategy reports, these data establish that the process of active updating in running span is slow and cognitively demanding, making it difficult to use when presentation rates are fast.

Running span is a complex working memory (WM) task that places a unique demand on the maintenance of serially ordered information. It requires recall of the last *n* items in a list of unknown length. Participants appear to keep track of the relevant information by actively updating the target recall set in WM when new items are presented (Bunting, Cowan, & Saults, 2006; Hockey, 1973; Morris & Jones, 1990). There have been significant advances in identifying the component processes involved in tasks requiring the updating of semantic information in WM (Ecker, Lewandowsky, & Oberauer, 2014; Ecker, Oberauer, & Lewandowsky, 2014; Kessler & Meiran, 2008; Kessler & Oberauer, 2014; Lewis-Peacock, Kessler, & Oberauer, 2018; Oberauer, 2018). How serial order is updated in running span is less well understood. The aim of the present study was to provide the first fine-grained temporal analysis of the cognitive demands of the updating process in running span. This was achieved by using performance on a concurrent reaction time (RT) task to index the magnitude of cognitive demands across the course of a running span trial.

In a typical running span task, participants are presented with sequences of variable length and are asked to recall the last *n* items in serial order. Hockey (1973) identified two strategies that participants could use to perform the task: passive listening and active processing. The passive mode involves receiving incoming items without engaging in any additional processing or actively attempting to update the recall set. Cowan and colleagues proposed that incoming information could be stored as a sensory trace in the first instance and then converted to a categorical form appropriate for recall at the end of the list (Bunting et al., 2006; Cowan et al., 2005). One possibility is that with spoken presentation, participants could retrieve representations of the most recent list items from echoic memory, a form of temporary sensory storage to which all spoken inputs have obligatory access (Crowder & Morton, 1969). Consistent with previous studies, performance improved at faster rates of presentation when a passive strategy was adopted, possibly due to reduced susceptibility to time-based decay within echoic memory (Bunting et al., 2006; Hockey, 1973).

An active running span strategy involves encoding items while also updating the target recall set so that only the relevant *n* items are maintained in WM. Pollack, Johnson, and Knaff (1959) proposed that participants do this by dropping the old item from the target set and adding the new one. We have recently proposed that the highly unusual demands of keeping pace with such a high rate of change in memory items cannot be met by simply relying on established mechanisms within WM and that, as a consequence, participants must assemble a novel cognitive routine to perform the task (Gathercole, Dunning, Holmes, & Norris, 2019). This may involve the encoding of new items, repositioning of already encoded items, discarding items that no longer need to be retained, and storage and rehearsal (Postle, 2003; Postle, Berger, Goldstein, Curtis, & D’Esposito, 2001).

This drop-and-capture conceptualization of updating has been applied to tasks involving changes in the memory set determined by specific item features (e.g., Kessler & Oberauer, 2014, 2015). In these tasks, a single item is identified as no longer relevant on the basis of a particular spatial location or semantic category and has to be modified in some way or replaced by a new item. Mechanisms proposed to support this updating process include item removal (Ecker, Lewandowsky, et al., 2014; Ecker, Oberauer, et al., 2014; Kessler & Meiran, 2008; Kessler & Oberauer, 2014; Lewis-Peacock et al., 2018), inhibition of irrelevant items (Hasher & Zacks, 1988; Hasher, Zacks, & May, 1999; Jonides, Smith, Marshuetz, Koeppe, & Reuter-Lorenz, 1998), and attentional shifts away from no-longer relevant representations (Cowan, 2001; McElree, 2001). These accounts all assume that updating would only alter the to-be-updated item, leaving the representations of the remaining items and their positions unchanged.

The distinctive challenge of running span is changing the position associated with each item in the target set as a new item is presented (Chatham et al., 2011; Juvina & Taatgen, 2007; Postle et al., 2001). Exactly how this might be achieved depends on the assumptions made about how serial order is encoded in the first place. In one class of models, order is represented by pairwise associations between items and a representation of order, such as a slowly evolving temporal context (e.g., Burgess & Hitch, 1992, 1999; see also, Oberauer, Lewandowsky, Farrell, Jarrold, & Greaves, 2012). One possibility is that in running span, these associations are changed as each new item arrives through a series of unbindings and rebindings to each of the *n* positions. The need for multiple rebindings can be avoided in models that represent sequences in terms of relative item order rather than absolute positions. An example is the primacy model by Page and Norris (1998). This represents serial order as an activation gradient that diminishes across successive list positions and where items are retrieved in order of their activation levels. After encoding *n* items in running span, each subsequent item could trigger an update process to suppress the most active (earliest) item. The second item would then have the highest activation becoming the first item to be recalled. Another order-based approach involves associating items with a cyclical representation of a temporal context. Chatham et al. (2011) developed a computational model of the *n*-back task employing a ring of *n* context-item associations. As each new item arrives the earliest context-item association is rebound to the new (latest) item. In principle, the same approach could be applied to running span and Chatham et al. (2011) reported that they had successfully done so (p. 3609). In this case, recall would simply commence by reinstating the context from the position subsequent to the final encoding position at the end of the list and then stepping through the remaining context cues until *n* items have been recalled.

Some characteristics of running span with implications for how serial updating may be accomplished are already known. First, active updating in running span appears to be a time-consuming process. Hockey demonstrated that when participants were instructed to use an active strategy, recall accuracy improved as the rate of presentation was slowed (Hockey, 1973). This recall benefit emerged even in the absence of explicit strategy instructions (Bunting et al., 2006). The clear implication is that updating takes time and cannot be applied as effectively with rapid presentation rates. Second, updating is highly cognitively demanding. Participants report that slow-paced running span associated with active updating is more challenging than a fast-paced task involving passive listening (Bunting et al., 2006). Morris and Jones (1990) found that performance accuracy was superior for short lists that required no updates of the target recall set compared with longer sequences that did require updates. This suggests a specificity of the updating effect to lists longer than the target recall set. The same study also found that recall was independent of the number of updating steps (Jahanshahi, Saleem, Ho, Fuller, & Dirnberger, 2008; Postle et al., 2001; Ruiz & Elosúa, 2013; Salthouse, 2014). This indicates that the resources required to support updating might be independently deployed and replenished within each interstimulus interval (ISI; 1-s duration in the case of the study by Morris & Jones, 1990). This may reflect the resource recovery rate for updating (Morris & Jones, 1990; Postle et al., 2001).

In summary, with rapid presentation rates there is inadequate time in running span tasks to update and participants opt for passive listening. When updating does occur, it increases recall accuracy and consumes resources but, with a sufficiently long interval between items, the depleted resources may be restored in time for the next updating epoch. The current experiments investigated the temporal characteristics of this resource demand at a finer level of detail, both within the presentation interval for each item and across the course of the trial as a whole. Experiment 1 examined the time course of cognitive demands in a slow-paced running span, comparing it to a serial recall task with no updating (simple span) and another requiring the encoding of a completely new sequence (modified span). Experiment 2 investigated the extent to which temporal characteristics of running span identified in Experiment 1 are restricted to the slow rates of presentation associated with active updating.

Both experiments assessed the demand on general cognitive resources in running span using a divided attention method (Craik, Govoni, Naveh-Benjamin, & Anderson, 1996). This method assumes that if two tasks are performed together and given equal priority, they should compete for the same general cognitive resources. The resources available for each task should therefore diminish, generating a dual task cost detectable in increased RTs to the concurrent task. A recent study applied this method by adding a concurrent choice RT (CRT) task between stimulus presentation and recall with the aim of examining the cognitive demands of different maintenance strategies for verbal items (Thalmann, Souza, & Oberauer, 2019). In our experiments, participants engaged in a similar CRT task during stimulus presentation in running span and two comparison serial recall tasks. This allowed us to examine the RT costs of time-locked events during stimulus presentation to identify the magnitude and time course of the resource demands specific to running span.

## Experiment 1

In line with previous findings, it was anticipated that a slow presentation rate would encourage the use of an active updating strategy in running span (Bunting et al., 2006; Hockey, 1973). The continuous demand metric from the concurrent CRT task allowed us to investigate whether the predicted level of high demand remains constant over the course of a running span episode or varies as a function of within-trial events. An invariant, trial-wide RT cost could provide evidence for a general resource-intensive mode in running span that continuously monitors and updates serial order. Alternatively, additional resources may be required only when the length of the encoded list exceeds the target number of items to be recalled (Morris & Jones, 1990; Postle et al., 2001). In this case, a heightened demand would be expected specifically during items in the list from *n* + 1th position onward, where *n* is the number of target items to be recalled.

In this experiment the resource demands of running span were compared with two other serial recall tasks. The first is simple span, a standard serial recall task that does not require updating of the encoded memory items. Updating in running span was expected to impose a greater cognitive demand than simple span. An absence of any differential demand between running span and simple span would suggest the use of a passive strategy in the former. The second comparison task is a modified version of simple span in which each trial contained successive lists of seven items with only the latest list relevant for recall at any given point. Tone cues were presented to cue participants to either recall the latest encoded list or to disregard prior items and start encoding a new list. Including this task allowed us to distinguish the possible costs of a periodic and complete reset of memory (in modified span) with a continuous and partial update (in running span). Previous studies of item-wise updating found that participants responded faster when an entire set of encoded items was to be changed compared with trials when only a subset of the encoded set was to be changed (e.g., Ecker, Oberauer, et al., 2014; Kessler & Meiran, 2008). It has been argued such a complete memory wipe can be achieved in a rapid, single process such that updating latencies only reflect the time taken to encode the new items (Ecker, Oberauer, et al., 2014). Notably, the complete memory update in the previous studies proceeded using simultaneous presentation of the new target set. This was different from the update in modified span in the present study as we used sequential presentation of target items. Here, the alerting tone and the first item of the subsequent list together cued participants to disregard previous items (update) while the remaining items were subsequently encoded one at a time.

Following previous findings that complete updates are less challenging than partial updates, it was speculated that modified span would impose less demand on cognitive resources than running span. A comparison of simple and modified span also allowed us to examine possible costs of a shift from maintaining a memory set to encoding an entirely new one. Recall performance was equated across the three memory tasks by using different numbers of target items to be recalled based on pilot data. Differential task difficulty could therefore be ruled out as a source of task differences in concurrent RTs.

### Method

#### Participants

Ninety-two native English speakers participated in the study. Complete data were recorded for 90 participants (68 females, *M*_age_ = 24.38 years, *SD* = 4.04 years). Participants were recruited using printed and electronic advertisements within and beyond the Medical Research Council Cognition and Brain Science Unit’s research participation system. Informed consent was obtained in accordance with ethical approval from Cambridge Psychology Research Ethics Committee (PRE 2016.066), and participants were compensated for their time and travel costs.

#### Design

The study used a 3 × 2 mixed factorial design. Task was a between-groups factor; participants were randomly assigned to one of three groups completing different memory tasks (simple span, modified span, and running span). These tasks are termed the WM tasks. Attentional load was a within-subject factor, with each participant completing two load conditions (single and dual load). In addition, all participants also completed a digit span task for an assessment of verbal short-term memory (STM) capacity.

An a priori power analysis for the present study was not possible, as there was little previous research using the divided attention paradigm with such fine calibration. Our choice of a sample size of 30 per group was informed by previous studies investigating running span (Bunting et al., 2006; Ruiz & Elosúa, 2013). A more recent study using divided attention to probe memory processes also used a similarly sized sample (Thalmann et al., 2019).

#### Procedure

For each participant, the digit span task was administered first, followed by the three experimental tasks: the CRT task, the WM task (running span, simple span, modified span), and the dual load condition in which both CRT and WM tasks were performed concurrently. These were implemented using a blocked design with a fixed order (CRT, WM, and dual task) repeated over one practice block and five experimental blocks. All tasks were completed within one session, typically lasting between 1.5 and 2 hr. All tasks were designed and presented on a PC using MATLAB 2014a (Mathworks, Natick, MA) and Psychtoolbox-3 (Brainard, 1997; Kleiner et al., 2007; Pelli, 1997).

#### Memory tasks

**Figure 1 fig1:**
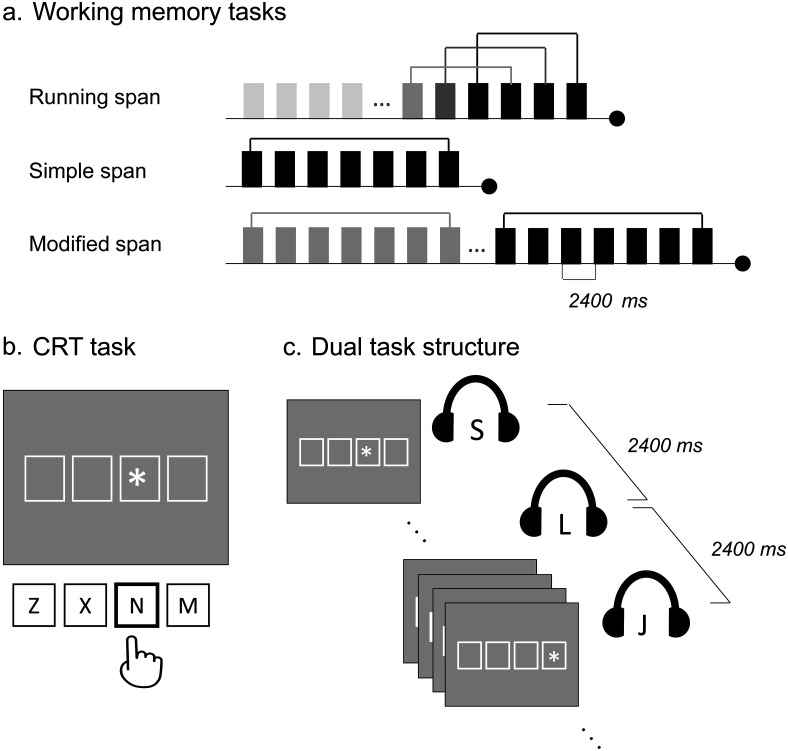
Task design. (a) Schematic of a memory list for each working memory task. Memory items (marked in rectangular boxes) were presented sequentially at a rate of 2,400 ms per item (800 ms for item, followed by 1,600 ms of silent interval) using spoken presentation. List length varied in modified span and running span. In long lists, the later items in the list were relevant for recall (marked in black, varied as per task), while earlier items were not (gray). The brackets above items denote the items in the same target recall set at a given time point. See text for further task descriptions. (b) The continuous choice reaction time (RT; CRT) task. Four square frames were presented on screen corresponding to four response keys. Participants pressed the key corresponding to the frame containing the star. A CRT stimulus was presented immediately following the response to the previous stimulus. (c) Dual task structure with a simultaneous application of (auditory) working memory and (visual) CRT task. Memory items presented every 2,400 ms; CRT task was participant paced, so the number of CRT stimuli presented between each memory item varied contingent on participant RTs. CRT = choice reaction time.

Three WM tasks (Figure 1a) were completed by separate groups of participants. The task instructions, length of lists, and number of target items varied across tasks as detailed below. The number of to-be-recalled items within each task was determined on the basis of pilot data; different sizes of target recall sets were tested, and the target recall set associated with an accuracy between 75 and 85% in each memory task was selected. Participants’ recall was digitally recorded using a microphone on a headset and subsequently transcribed. Recall accuracy was measured in terms of the proportion of items recalled in the correct serial position.[Fig-anchor fig1]

##### Running span

Participants attempted to recall the last four items of the presented list in correct serial order. Lists were preceded by a 1-s tone and contained four to 12 items. The length of the list was unknown to participants. Each block contained 10 trials, including one presentation of each list length, except for lists containing eight items that were presented twice in a pseudorandom order. Participants thus completed 50 trials over five blocks.

##### Simple span

Each list contained seven items and participants attempted to recall all items in order. Fifty lists were presented over five blocks.

##### Modified span

Participants were presented with sequences of letters periodically interspersed with tone cues that indicated the start of the set of items they were to remember. The sequences contained seven, 14, 21, or 28 items. Therefore, each sequence to be recalled within the longer lists comprised one to four target sets of seven items. Items in Positions 8, 15, and 22 were considered update items as they were presented in the context of longer lists. Even though they represented the start of a memory list, this differed from the item at Position 1. While Item 1 was always the start of a new list, update items were the start of later sequences, preceded by one or more target sequences. A 1-s tone was presented before each update item to alert the participants to start encoding a new set of items. Participants were asked to recall the last target set immediately following the end of the sequence. The number of items to be recalled was always fixed at seven, as in simple span, but the length of each list was unknown to the participants. Each list length was presented twice in a pseudorandom order in a block. There were 40 trials presented over five blocks.

##### List generation

Identical protocols were employed to present stimuli and generate sequences in all three memory tasks. Lists were generated randomly subject to the following constraints: (a) 20 consonants were used as stimuli (*W* was excluded), (b) a letter could only be repeated after every seven items, (c) two phonologically similar letters could not be presented consecutively, and (d) three or more letters could not be presented in alphabetic order. A typical list of nine items was *D, S, P, Y, R, L, G, K, D.*

The letters were spoken by a male British English speaker and were recorded at a sample rate of 44.1 kHz. Sound files containing each letter were edited to be 800 ms long and the location of the letter within that duration was adjusted so that letters sounded evenly spaced (Morton, Marcus, & Frankish, 1976). The tasks used auditory presentation over headphones (Sennheiser HD 280 PRO II, Sennheiser electronic GmbH & Co., Tullamore, Ireland) at a rate of 2,400 ms per item (i.e., 800 ms for item presentation followed by a silent interval of 1,600 ms). Each list was preceded by a 1-s tone to alert participants to the start of the trial and a fixation cross was displayed on the screen throughout the trial. The duration of the presentation phase varied with list length and was always immediately followed by spoken serial recall for a maximum of 20 s.

#### CRT task

In the CRT task (Figure 1b), an asterisk was presented in one of four possible square frames on the screen, and participants were required to press the key corresponding to the frame containing the asterisk as quickly and accurately as possible. Following a keypress, the asterisk immediately shifted from its current frame to one of the other three frames chosen at random. Participants responded using the first two fingers of both the right and left hands. The task was self-paced and continuous, with onset of the next stimulus immediately following each response. Both RT and accuracy were recorded in this task.

#### Dual load condition

In the dual load conditions (Figure 1c), participants simultaneously performed the WM and CRT tasks and were instructed to treat each task with equal priority. The presentation protocol and list and stimulus generation in the dual task conditions were identical to their corresponding single load conditions. The visual presentation of the first stimulus in the CRT task was synchronized with the auditory onset of the memory sequence in the respective WM task. Thereafter, the CRT stimuli were presented successively during the presentation of the memory list and ceased when the retrieval phase of the WM task started. Participants were instructed to respond in the CRT task as quickly and accurately as possible while also attending to the memory list for subsequent recall in the respective WM task.

#### Digit span task

In addition to the tasks above, a digit span task was administered to measure verbal short term memory capacity. For this, digits 0 to 9 were presented sequentially in the center of the screen for 1,000 ms followed by a blank ISI of 1,000 ms. Stimuli were pseudorandomly selected such that stimulus repeats were only allowed after every nine items. At the end of the list, the digits were presented in a grid on screen and participants recalled the presented sequence in serial order by indicating responses with mouse clicks. The task commenced with lists containing four items. At each span level, four trials were presented. If participants responded with 75% accuracy at any given level, they advanced to the next level (i.e., the list lengthened by one item). The task ended once performance failed to meet this condition. Span was recorded as the longest list length at which participants correctly recalled three or more trials.

#### Analysis plan

##### RT

Responses occurring faster than 200 ms after CRT stimulus onset or due to accidental holding down of a response key from the previous trial were excluded in both single- and dual-load conditions of the CRT task (Van Zandt & Townsend, 2014). Programming constraints caused the CRT task to abruptly stop upon reaching the end of the memory list in the dual load condition, which truncated the recording of any CRT responses that may have followed. Therefore, dual CRT responses associated with the final item in each memory list across all tasks were removed. The data were then trimmed by first removing inaccurate responses; CRTs that deviated from individual means by more than 2.5 standard deviations and individuals who deviated from the respective group mean by more than three standard deviations were removed.

The experiment was designed to test the prediction that active updating in running span would demand greater cognitive resources compared with simple serial recall. No strong hypotheses were made regarding the temporal characteristics of the resource demands within the trials. The statistical analyses reflected this exploratory approach. At the task level, the difference between single and dual CRTs across the three memory tasks was examined using a 2 × 3 analysis of variance (ANOVA) with two factors: load (single vs. dual) and memory task (running span, modified span, and simple span). At the trial level, the difference in dual CRTs between early and late list positions was examined across the three tasks. For this, CRTs were partitioned into early positions (1 to 4) and late positions (5 and 6) to test if the effect of updating was found across the course of a running span list or specifically from items *n* + 1 onward with *n* = 4 in running span. CRTs associated with later items only included Items 5 and 6 rather than all later updating items in running span. This was because these were the only updating items in running span that could be compared with items at the same positions in the other tasks, as simple span did not have later list positions. This was analyzed using a 2 × 3 ANOVA with two factors: position (early vs. late) and memory task. To investigate the variation in dual CRTs during the interval between items, the interitem interval of 2,400 ms was divided into six bins of 400 ms each, with two bins of stimulus presentation and four bins of silent postpresentation interval. This was analyzed using a 6 × 2 × 3 ANOVA with three factors: bin (six 400 ms bins), position (early vs. late, separated at position four in the list as described above), and memory task. A similar analysis was also carried out to compare variation in dual CRTs at the item level specifically between updating items in running span and modified span. This requires a comparison of dual CRTs at Item 5 onward in running span and Items 8, 15, and 22 in modified span as these were the items at which memory updating was required.

##### Recall

Performance in the memory tasks was scored as the proportion of items recalled in their correct serial position. The data were screened for outliers deviating by more than three standard deviations from the group mean (none detected). The effects of load and target position on recall accuracy were investigated separately for each task. For this, three ANOVAs were used to test if there was a difference in recall accuracy between single- and dual-load conditions across the target positions (four in running span, seven in both simple span and modified span).

Post hoc tests were used to explore significant interactions terms for both RT and recall analyses. The results from these post hoc tests are summarized in the main text and reported in detail in the online supplemental materials available online. Bonferroni correction for multiple comparisons was applied and both corrected and uncorrected *p* values are presented. A Greenhouse-Geisser correction was applied in case sphericity was violated in factors with repeated measures.

### Results

Table 1 provides participant characteristics and overall task performance in both CRTs and recall accuracy across both load conditions. There were no group differences in age, *F*(2, 87) = 2.14, *p* = .12, gender, χ^2^(2, *N* = 90) = .12, *p* = .94, or verbal STM capacity, *F*(2, 87) = 0.21, *p* = .81.[Table-anchor tbl1]

#### RT

**Figure 2 fig2:**
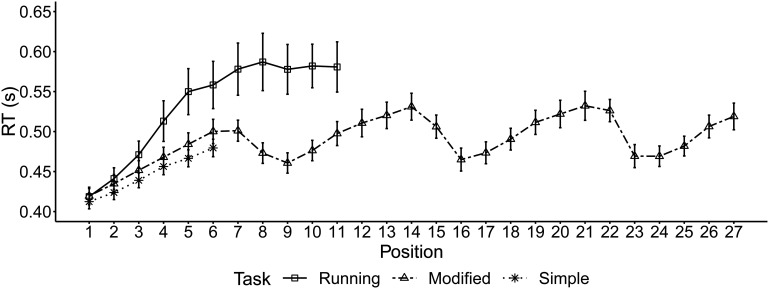
Concurrent choice reaction times (RTs; CRTs) across list positions for each memory task. Note that the data are averaged across all list lengths, thus later positions in running and modified span contribute fewer data points. RTs associated with the final position across lists are not displayed here, see text for data exclusion. Error bars represent standard error of the mean.

**Figure 3 fig3:**
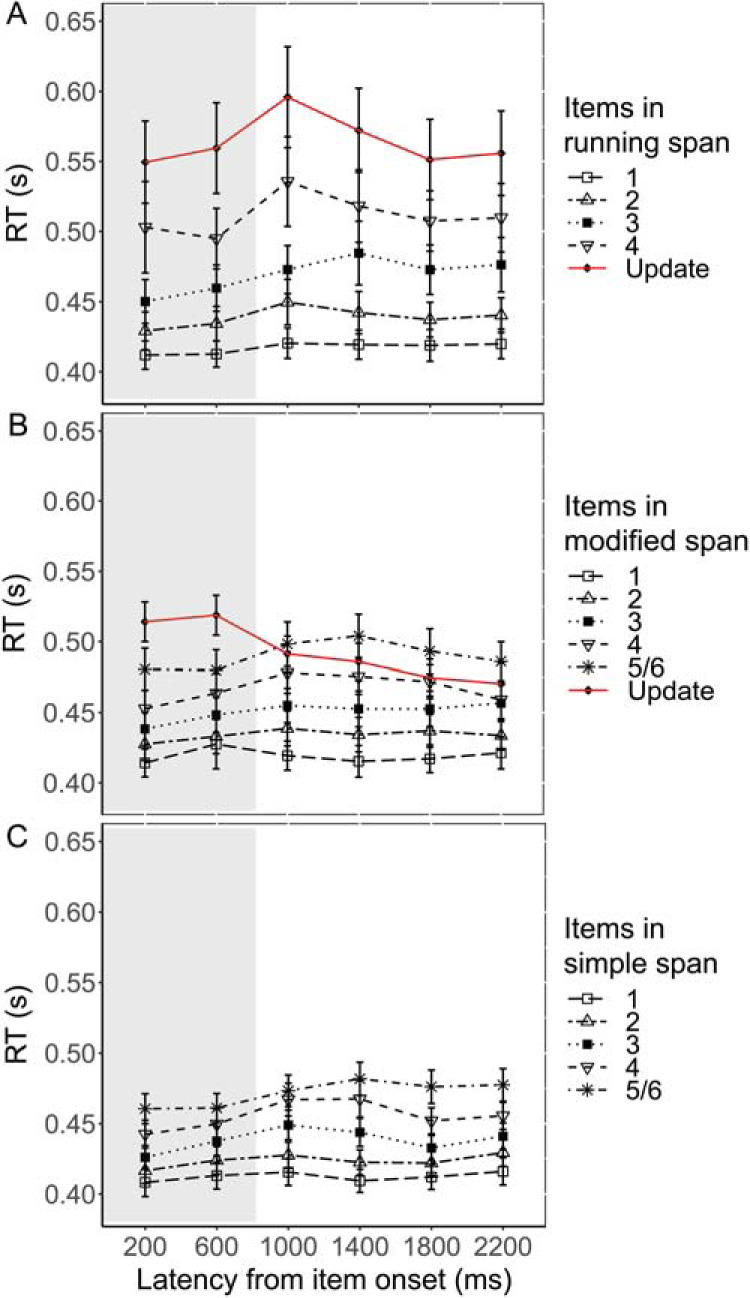
Concurrent choice reaction times (CRTs) as a function of latency from item onset of memory item, plotted separately for items. Please note that while the data are illustrated per position, the analysis collapsed items into early and late positions (see text for more). The first 800 ms represent the duration of the item presentation (shaded in gray), followed by a 1,600 ms silent interitem interval (unshaded). (A) CRTs within running span, with Positions 1, 2, 3, and 4 (black lines) and update items from Position 5 onward (red line). (B) CRTs within modified span, with Positions 1, 2, 3, 4, and 5/6 (black lines) and update items at positions 8, 15, and 22 (red line). (C) CRTs within simple span, with Positions 1, 2, 3, 4, and 5/6 (black lines) with no update items in the task. Error bars represent standard error of the mean.

Two participants were removed after outlier screening, leaving 29 participants each in running span and simple span and 30 in modified span. The data from the CRT task under dual load are illustrated in Figure 2 (across trials) and Figure 3 (across bins at the item level).[Fig-anchor fig2][Fig-anchor fig3]

##### Task-level analyses

CRTs in the single- and dual-load conditions were compared across the three groups completing different memory tasks using a 2 × 3 ANOVA. There was a main effect of load, such that responses in the single-load condition were faster than in the dual-load condition, *F*(1, 85) = 72.84, *p* < .001, η_*p*_^2^ = .46. There was also a significant interaction between load and memory task, *F*(2, 85) = 5.20, *p* = .007, η_*p*_^2^ = .11. This interaction was explored using post hoc tests (Table S1 in the online supplemental material) which showed that single CRTs did not differ between tasks. In contrast, dual CRTs in simple span were faster than both running span, *t*(56) = 2.22, *p* = .03, mean difference = 43 ms, and modified span, *t*(57) = 2.07, *p* = .04, mean difference = 33 ms, but dual CRTs were not significantly different between running span and modified span, *t*(57) = .47, *p* = .64.

##### Trial-level analyses

Dual CRTs during early positions (1 to 4) and late positions (5 and 6) were compared across the three memory tasks using a 2 × 3 ANOVA. There was a significant effect of position, *F*(1, 85) = 56.22, *p* < .001, η_*p*_^2^ = .40, as well as a significant interaction between position and task, *F*(2, 85) = 7.60, *p* = .001, η_*p*_^2^ = .15. Post hoc tests (Table S2 in the online supplemental materials) showed that CRTs during early positions were not significantly different between any pair of memory tasks, while those during late positions in running span were significantly slower than simple span, *t*(56) = 2.57, *p* = .01, mean difference = 80 ms. The CRTs during late positions in modified span were not significantly different from simple span or running span, *p*s > .05.

##### Item-level analyses

CRTs across the six bins of the item interval (400 ms each) were compared between early and late positions and across three memory tasks in a 6 × 2 × 3 ANOVA. Table S3 (available in the online supplemental materials) presents the results from the omnibus ANOVA. There was a significant three-way interaction between bin, position, and task, *F*(7.35, 308.2) = 2.56, *p* = .01, η_*p*_^2^ = .06. To explore the interaction, two post hoc 6 × 3 mixed measure ANOVAs were conducted to examine the interaction between bin and task separately for early and late positions. During early positions there was no interaction between bin and task, *F*(6.0, 255.1) = 1.11, *p* = .36, η_*p*_^2^ = .03, while during late positions, a significant interaction was found, *F*(6.3, 269.6) = 3.03, *p* = .006, η_*p*_^2^ = .07. Further post hoc tests (Table S4 in the online supplemental materials) showed that during these late positions, there was an increase in CRT from the second to third bin (centered around 1,000 ms) for all three tasks. The magnitude of this peak was greatest in running span, *t*(28) = 3.65, *p* = .001, mean difference = 40 ms, compared with modified span, *t*(29) = 3.18, *p* = .003, mean difference = 18 ms, and simple span, *t*(28) = 3.36, *p* = .002, mean difference = 12 ms. Also, this CRT peak at 1,000 ms was specific to running span and was not found during update items in modified span, showing that it was not just common to any WM updating event (Table S5 in the online supplemental materials). In fact, in modified span there was a CRT dip at the same time point, as illustrated in Figure 3.

#### Recall

**Figure 4 fig4:**
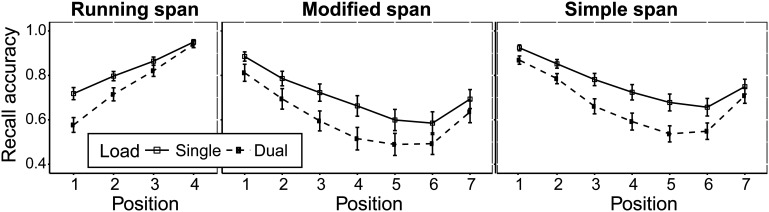
Recall accuracy (proportion of items recalled in correct serial position) across each serial position for the three memory tasks across single (solid line) and dual (dashed line) load conditions. Error bars represent standard error of the mean.

The recall accuracy data from the memory tasks are illustrated in Figure 4. Performance under single and dual loads was compared across target positions separately for each task using repeated measures ANOVAs. Post hoc paired-sample *t* tests are presented in Table S6 (available in the online supplemental materials).[Fig-anchor fig4]

In running span, there was a main effect of load, *F*(1, 29) = 22.19, *p* < .001, η_*p*_^2^ = 0.43, and target position, *F*(1.5, 44.4) = 121.23, *p* < .001, η_*p*_^2^ = 0.80. There was also a significant interaction between load and position, *F*(2.3, 66.4) = 19.68, *p* < .001, η_*p*_^2^ = 0.40, with the effect of load decreasing across successive recall positions.

Recall in modified span exhibited a significant effect of both load, *F*(1, 29) = 47.59, *p* < .001, η_*p*_^2^ = 0.62, and target position, *F*(2.7, 76.9) = 53.82, *p* < .001, η_*p*_^2^ = 0.65. Load and position also showed a significant interaction, *F*(3.3, 94.4) = 3.55, *p* = .002, η_*p*_^2^ = 0.11, such that the effect of load on recall increased from serial Positions 1 to 4, and then decreased from Position 5 onward.

Similarly, recall in simple span showed a significant effect of load, *F*(1, 29) = 53.29, *p* < .001, η_*p*_^2^ = 0.65, and target position, *F*(2.2, 62.4) = 57.03, *p* < .001, η_*p*_^2^ = 0.66. Load and position also showed a significant interaction, *F*(4.4, 128.4) = 8.61, *p* < .001, η_*p*_^2^ = 0.23, such that the effect of load on recall increased from serial Positions 1 to 5 and decreased from Positions 6 to 7.

### Discussion

In this experiment the time course of the cognitive demands of three serial recall tasks were assessed by their impact on concurrent task RTs. The RT data showed differential patterns across the three memory tasks. Running span, relative to simple span, was associated with slower RTs at later list positions. RTs peaked 1,000 ms from item onset at these later positions and this peak was larger in running span than the other memory tasks. Also, this peak was not present for update items in modified span.

The RT costs of running span were evident from the presentation of *n* items to the end of the sequence. This profile indicates that the complexity of the processes involved in the running span tasks increases after position *n* once simple serial recall of the presented items is no longer sufficient. This conclusion is consistent with other proposals that updating involves of a set of processes that are applied only when necessary rather than representing a broader mode of processing adopted over the full course of a running span trial (Chatham et al., 2011; Juvina & Taatgen, 2007). At the fourth (= *n*th) serial position, the demand profile resembled that of later items. This might indicate an anticipatory process wherein participants prepare for the upcoming update event, perhaps by already recoding the stored target set or even starting to preemptively update their target set.

Closer examination revealed that the RT costs were continuously elevated at positions *n* + 1 onward, rising to a peak after about 1,000 ms from item onset. By the time the next item was presented, the costs had returned to baseline levels. This time course is consistent with a previous suggestion that individual episodes of updating do not impose cumulative demands on cognitive resources (Morris & Jones, 1990; Postle, 2003; Postle et al., 2001). On the basis of the present data we do not know whether the speed of these cognitive processes involved in updating is fixed or can be modulated to fit the temporal parameters of the task. A lower peak in RT costs was also observed in simple span and modified span at the 1,000 ms time point. These costs may reflect item maintenance processes such as rehearsal or attentional refreshing likely to be common to all serial recall tasks (Barrouillet, Bernardin, & Camos, 2004; Barrouillet, Gavens, Vergauwe, Gaillard, & Camos, 2009; Towse, Hitch, & Hutton, 1998). The greater magnitude of the cost in running span relative to the other two tasks can be attributed to the additional processes involved in updating. It should be noted though that the differential cost function for running span cannot be simply explained in terms of task difficulty as recall accuracy was comparable for all three memory tasks.

The cognitive demands of the cued memory reset in modified span were distinct from the internally driven, serial update of already encoded items that takes place in running span. The time course of the RT costs of modified span were cyclical, increasing with each new item in the seven-item sequence (as in simple span) and returning to baseline after the encoding of each new set, if restarted, at Positions 8, 15, and 22. The RT function during the interval of these update/reset items was very different from that in running span, with demand diminishing rather than rising to a peak following item offset. The data suggest that reinitializing encoding midsequence has low cognitive demands and is equivalent to starting at the beginning of a list. This is in line with previous observations that a complete memory reset is faster than a partial update of the target set (Kessler & Meiran, 2008). It is also reinforced by the equivalent serial position functions in modified span and simple span.

If the 1,000 ms peak in demand in running span reflects updating, it should diminish under conditions when an active strategy is not adopted. Experiment 2 tested this prediction by manipulating the time available for participants to adopt an active strategy. Previous investigations showed that when faced with a faster rate of presentation, participants shift to passive listening, a strategy that demands fewer resources during item presentation (Bunting et al., 2006; Cowan et al., 2005; Hockey, 1973). Fast presentation rates should therefore reduce active updating and decrease the demand associated with running span.

## Experiment 2

It is well-established that presentation rates of two or more items per second favor a passive strategy in running span while rates of one or fewer items per second encourage the use of an active updating strategy (Botto, Basso, Ferrari, & Palladino, 2014; Broadway & Engle, 2010; Bunting et al., 2006; Collette et al., 2007; Cowan et al., 2005; Elosúa & Ruiz, 2008; Hockey, 1973; Kiss, Pisio, Francois, & Schopflocher, 1998; Morris & Jones, 1990; Postle, 2003; Postle et al., 2001; Ruiz & Elosúa, 2013). The performance benefits associated with active updating progressively increase as the rate slows (Hamilton & Hockey, 1974; Hockey, 1973).

Experiment 2 tested the proposal that the time course of demand under divided attention in Experiment 1 reflects an active updating process. If this is the case, the distinct temporal signature of an elevated cost peaking 1,000 ms after item onset should be eliminated at presentation rates faster than one item per second. Experiment 2 compared running span at three presentation rates—fast (400 ms/item), medium (800 ms/item), and slow (1,600 ms/item). The intermediate rate was selected as a midpoint between the rates associated with the active and passive strategies in which we expected to see a hybrid profile reflecting mixed active and passive strategies.

Each running span condition was combined with the CRT task. We aimed to test our prediction that CRTs would increase as participants shifted from passive listening to active updating (from fast to slow rates). An additional aim of this experiment was to establish whether the time course of updating can be modulated by presentation rate. The slow presentation rate in this experiment was faster than that employed in Experiment 1 (1,600 ms/item as opposed to 2,400 ms/item). If the rate of updating can be increased when required, the resource burst detected at 1,000 ms after item onset in Experiment 1 might occur earlier in time during the slow rate in Experiment 2. If updating imposes a time-invariant load though, its timing would be preserved across both experiments. The inclusion of the medium rate condition also provided the opportunity to examine whether the process of updating could be speeded when presentation was faster than one item per second.

### Method

#### Participants

Thirty native English speakers were recruited for this experiment (18 females, *M*_age_ = 24.3 years, *SD* = 3.9 years). As the observed power in Experiment 1 was greater than .95 for most analyses, we chose the same sample size (30) for this experiment. Note that in the first experiment, 30 participants completed only one of three tasks in a between-groups design. Here, all 30 participants completed each of the three conditions in a within-subject design. The experiment was approved by the Cambridge Psychology Research Ethics Committee (PRE 2016.066), and participants were compensated for their time and travel costs.

#### Design

This experiment used a 3 × 2 within-subject design to investigate two factors: presentation rate and attentional load. Participants completed running span with different rates of item presentation (fast, medium, and slow) counterbalanced across sessions on different days. Attentional load was also manipulated. Performance was measured under both single- and dual-load conditions in each session.

#### Procedure

Participants attended three sessions in this experiment. In each session, they completed three tasks: the CRT task, the running span task (with presentation rates applied in a counterbalanced order across sessions), and the dual load condition in which both tasks were performed concurrently. Task order was fixed (CRT, running span, and dual task) in each block and an initial practice block was followed by five experimental blocks. Each session lasted approximately an hour and concluded with the administration of a strategy questionnaire.

#### Task

The structure of the running span task employed in this experiment differed from that in Experiment 1 in two respects. First, a new set of stimuli were recorded so that the presentation of the memory items (i.e., letters) lasted for 400 ms. For this, letters spoken at a rate of two letters per second by a native British English female speaker were recorded. Using the P-center approach (Morton et al., 1976), the audio was segmented into constituent letters and then compressed into 400 ms files using Adobe Audition 3.0. Second, the interitem interval in the task varied across the rate conditions. The fast-paced task involved successive presentations of items for 400 ms with no intervals between them. In the medium rate condition, a silent 400 ms interval was interleaved between successive items, such that items were presented at a rate of 800 ms/item. The silent interval was increased to 1,200 ms in the slow rate condition making the presenting rate 1,600 ms/item. All other features of the task including modality of presentation and recall, list generation protocol, and number of trials and blocks in each session were the same as in Experiment 1.

#### Strategy reports

At the end of each session participants were provided with a list of six strategies adapted from Norris, Hall, and Gathercole (2019). The strategies were: (a) passively receive the letters, (b) rehearse the letters as they were presented, (c) keep up with the last four letters as they were presented, (d) group the letters by separating them into sets of particular sizes, (e) group the letters according to their meaning, and (f) form a mental image of the letters. Participants were asked to rate the frequency with which they used each strategy on a 4-point scale ranging from 0 (*almost never*) to *occasionally* to *frequently* to 3 (*almost always*). Further, at the end of the final session, participants were asked to report the rate condition they experienced as the least challenging.

#### Analysis plan

The CRT and recall data were trimmed and outlier screening and correction proceeded as in Experiment 1.

##### RT

A confirmatory approach to analysis was used in this experiment as it was designed to test hypotheses derived from the results of Experiment 1. It was predicted that fast and slow presentation rates would be associated with differing levels of cognitive demand indexed by CRTs. At the task level, a 2 × 3 ANOVA examined the effect of two factors: load (single vs. dual CRTs) and rate of presentation (fast, medium and slow rates). An interaction between load and rate was predicted, such that dual CRTs would be larger for the slow than fast rate condition. At the trial level, a 2 × 3 ANOVA was used to examine the effect of list position (early items up to Position 4 (= *n*) vs. late items from Position 5 in the list) and presentation rate on dual CRTs. An interaction between position and rate was expected, such that the late CRTs would be larger for the slow than fast rate condition.

At the item level, the onset-to-onset interval (800 ms in the medium rate and 1,600 ms in the slow rate condition) was divided into smaller bins of 400 ms each, resulting in two bins in the medium rate condition and four bins in the slow rate condition. The item intervals in the fast rate condition were already 400 ms in duration and could not be divided further and were thus excluded from this analysis. In the medium rate condition, a 2 × 2 ANOVA examined the effect of position (early vs. late, as in Experiment 1) and bin (presentation vs. postpresentation bin). In the slow rate condition, a 2 × 4 ANOVA was similarly used to examine the effect of position (early vs. late) and bin (1 presentation bin and 3 postpresentation bins). A significant interaction between task and bin was expected in the slow rate and planned comparisons were used to test the timing of the anticipated peak. Assuming that the updating-related peak is time locked to 1 s from item onset, a CRT increase was anticipated from the second to the third bin during the late positions in the slow rate condition (replicating that in Experiment 1). If instead, the peak was found earlier in the interval, for example, between first and second bin, this would suggest that the updating process was rate sensitive. All analyses of CRTs in the medium rate condition were treated as exploratory as the relative use of an active strategy at this rate was unclear.

##### Recall

The effects of load, target position, and rate of presentation on recall accuracy were investigated in a 2 × 4 × 3 ANOVA to test if there was a difference between single and dual recall across the four target positions in the three rate conditions. Significant main effects of rate, position, and load were predicted and post hoc tests were used to explore interaction effects which are summarized in the Results section and detailed in the online supplemental materials.

##### Strategy use

Mean frequency ratings for the six strategies were compared across rate conditions in separate nonparametric Friedman tests.

### Results

Performance data from the running span and CRT tasks for both load conditions across the three presentation rates are presented in Table 2.[Table-anchor tbl2]

#### RT

**Figure 5 fig5:**
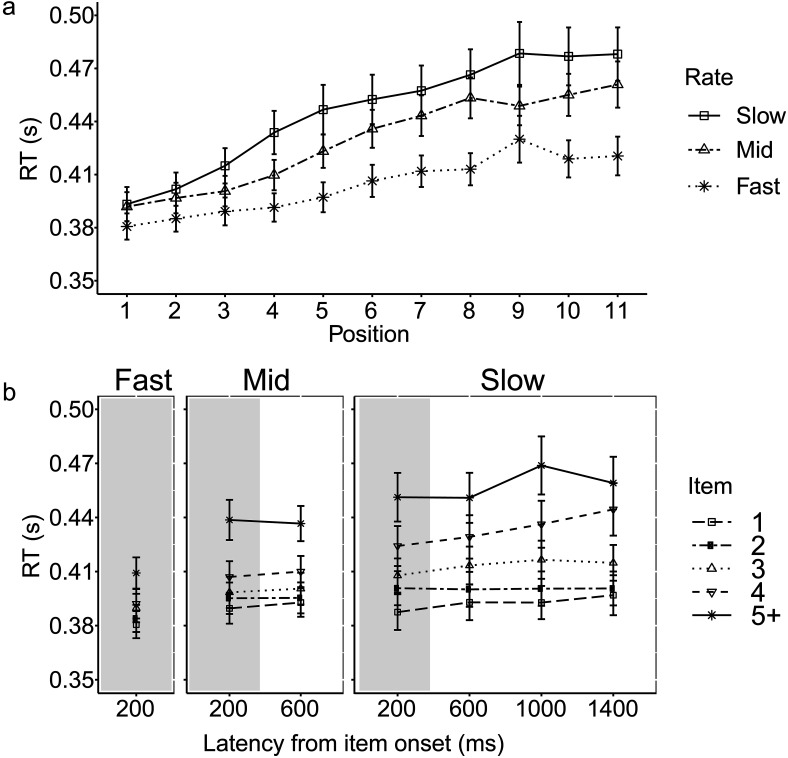
Choice reaction time (RT; CRT) data from Experiment 2. (a) Concurrent CRTs across list positions for each rate condition. Note that the data are averaged across all list lengths, thus later positions contribute fewer data points. RTs associated with the final position across lists are not displayed here, see text for data exclusion. (b) Concurrent CRTs as a function of latency from item onset of memory item for all three rate conditions, separated by Positions 1, 2, 3, and 4, and update items at Position 5 onward. Please note that while the data are illustrated per position, the analysis collapsed items into early and late positions (see text for more). The first 400 ms represent the duration of the item presentation (shaded in gray), followed by variable duration of silent interitem interval (unshaded). Error bars represent standard error of the mean.

One participant was removed after screening for outliers. The CRT data under dual load are presented in Figure 5a (across trials) and Figure 5b (across the item interval).[Fig-anchor fig5]

##### Task-level analysis

Single and dual CRTs were compared across the three rate conditions in a 2 × 3 ANOVA. There was a significant effect of rate, *F*(2, 56) = 3.72, *p* = .03, η_*p*_^2^ = .12, as well as a significant interaction between load and rate, *F*(2, 56) = 36.39, *p* < .001, η_*p*_^2^ = .57. Paired-sample *t* tests were conducted to compare single and dual CRTs between rate conditions (Table S7 in the online supplemental materials). As predicted, dual CRTs were substantially greater in the slow than fast rate condition, *t*(28) = 4.08, *p* < .001, mean difference = 33 ms. Dual CRTs in the medium rate were greater than in the fast rate, *t*(28) = 2.74, *p* = .01, mean difference = 17 ms, but lower than in the slow rate, *t*(28) = 2.21, *p* = .04, mean difference = 17 ms.

##### Trial-level analysis

Dual CRTs associated with early and late positions were compared across the three rates in a 2 × 3 ANOVA. There was a significant effect of both position, *F*(1, 28) = 76.59, *p* < .001, η_*p*_^2^ = .73, and rate, *F*(2, 56) = 8.69, *p* = .001, η_*p*_^2^ = .24, and a significant interaction between position and rate, *F*(1.6, 45.9) = 13.44, *p* < .001, η_*p*_^2^ = .32. At both early and late positions, the dual CRTs were significantly greater at the slow than fast rate, with a larger difference between rate conditions during the late positions, mean RT difference = 48 ms, *p* < .001, than early positions, mean RT difference = 22 ms, *p* = .005. The CRTs in the medium rate condition were greater than the fast rate condition but only during late positions, mean difference = 28 ms, *p* = .002 (see Table S8 in the online supplemental materials for further details).

##### Item-level analysis

In the slow condition, a 4 × 2 ANOVA compared CRTs in four bins (1 presentation and 3 postpresentation bins) and between early and late positions. There was a significant effect of bin, *F*(2.1, 56.2) = 9.85, *p* < .001, η_*p*_^2^ = .26, and position, *F*(1, 28) = 56.69, *p* < .001, η_*p*_^2^ = .68. In line with predictions, there was a significant interaction between bin and position, *F*(3, 84) = 7.17, *p* < .001, η_*p*_^2^ = .20. Planned comparisons showed that CRTs peaked around 1,000 ms from item onset during late positions, with no significant difference in the first two bins, an increase from second to third bin, mean RT difference = 18 ms, and a decrease from third to fourth bin, mean difference = 10 ms. There was no significant variation in CRTs across bins in early positions in running span (as detailed in Table S9 in the online supplemental materials).

In the medium rate condition, a 2 × 2 ANOVA examined CRTs in two bins (1 presentation and 1 postpresentation) between early and late positions. There was a main effect of position, *F*(1, 28) = 83.92, *p* < .001, η_*p*_^2^ = .75. The effect of bin was not significant, *F*(1, 28) = .001, *p* = .98, η_*p*_^2^ < .001, and there was no significant interaction between bin and position, *F*(1, 28) = 1.24, *p* = .28, η_*p*_^2^ = .04.

#### Recall data

**Figure 6 fig6:**
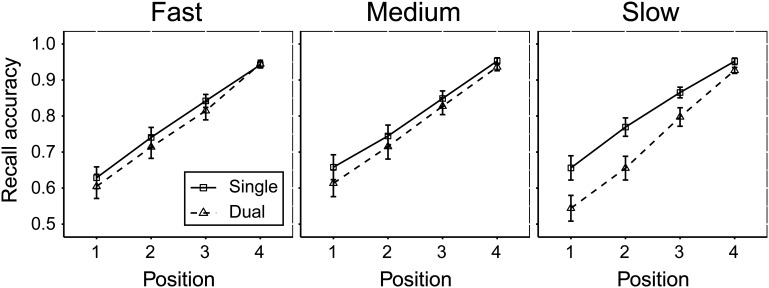
Recall accuracy (proportion of items recalled in correct serial position) across each serial position for the three rate conditions across single (solid line) and dual (dashed line) load conditions. Error bars represent standard error of the mean.

Recall data across the two loads conditions, four target positions, and three presentation rates are presented in Figure 6. A 2 × 4 × 3 ANOVA revealed a significant effect of load, *F*(1, 28) = 24.29, *p* < .001, η_*p*_^2^ = .47, and position, *F*(1.3, 34.7) = 126.84, *p* < .001, η_*p*_^2^ = .82, but no main effect of rate, *F*(2, 56) = 1.63, *p* = .21, η_*p*_^2^ = .06. There was a three-way interaction between load, position, and rate, *F*(6, 168) = 2.44, *p* = .03, η_*p*_^2^ = .08. This was explored using post hoc paired-sample *t* tests (Table S10 in the online supplemental materials). Dual recall was significantly lower than single recall in the slow rate condition across all positions, with greater dual-task impairment during the first two than the last two recall positions (all *p*s < .01). No significant difference between load conditions was found in the fast rate across all positions, (*p*s > .05), and the evidence suggested a trend in the medium rate condition in which dual recall was lower than single recall at early positions (uncorrected *p*s < .05), but not later positions in the recall set (*p*s > .05).[Fig-anchor fig6]

#### Strategy use

**Figure 7 fig7:**
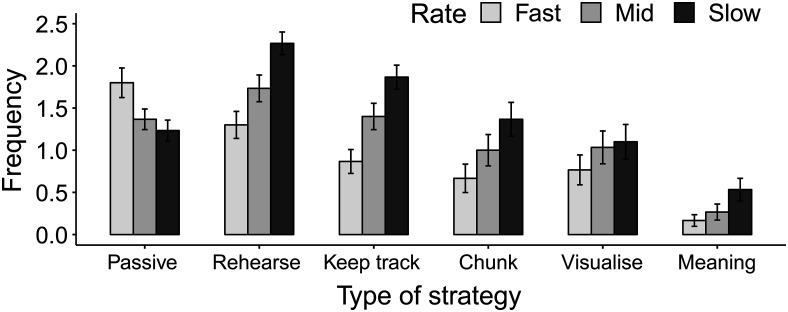
Mean frequency of self-reported strategy use for each rate condition. Participants rated their use of each strategy from 0 (*almost never*) to 3 (*almost always*); see text for strategy statements.

Participants reported the fast-paced condition as being the least demanding of the three presentation rates, χ^2^(2, *N* = 30) = 7.8, *p* < .05. The frequencies of strategy use are summarized in Figure 7. Overall, fewer strategies were reported at the fast presentation rate (0.93) compared with the medium (1.13) and the slow rates (1.39). The five active strategies were most frequently employed in the slow rate, followed by the medium and fast rate conditions. The exception to this pattern was passive listening, which was most frequently reported in the fast rate condition. Nonparametric Friedman tests showed that strategy use differed significantly across rates for all strategies, *p*s < .05 except for the use of mental imagery.[Fig-anchor fig7]

### Discussion

In this running span experiment, RTs on a concurrent CRT task increased with slower presentation rates. There was a marked difference in overall demand between fast and slow rates, with intermediate levels of costs at the medium presentation rate. CRTs increased across list positions over the course of a trial in all three rate conditions and the difference between fast and slow rates was more pronounced during late than early positions. These data indicate that the likelihood of engaging active processing increases with the interval between successive items. Consistent with this, participants reported the highest use of active strategies at the slowest presentation rate, preferring a passive listening strategy when presentation was the most rapid. This reinforces previous findings that the efficacy of instructed active or passive strategies varied with presentation rates (Bunting et al., 2006; Hockey, 1973). An unexpected result was that in the fast-paced condition, dual RTs were faster than single RTs. One possibility is that this reflects the strategic use of presentation rate as an external pacemaker to entrain CRT responding.

This experiment replicates the peak in RT at 1,000 ms after the onset of items beyond position *n* observed in Experiment 1, even though the slow presentation rate condition in Experiment 2 was faster than that employed in Experiment 1 by 800 ms per item. This indicates a fixed time course for updating rather than one paced by presentation rate. There was no comparable updating-related peak in the medium rate condition. It therefore appears that the 800 ms interval between consecutive onsets in the medium rate was too short for active updating. This explains why performance appears to suffer when participants attempt to adopt an active updating strategy at rates faster than 1,000 ms per item (Hockey, 1973).

Contrary to previous observations (Bunting et al., 2006; Hockey, 1973), presentation rate did not have an effect on either overall recall accuracy or serial position functions. However, the impairment in recall during dual load conditions did vary with rate. The dual task effect was significant only in the slow presentation rate across all positions and decreased with each successive position in the recall set. The division of resources between running span and the CRT task thus appears to impair performance disproportionately at slow presentation rates consistent with the view that an executively mediated update operation is specifically applied only during slow presentation.

## General Discussion

Two experiments tracked the cognitive demands of a verbal running span task requiring the serial recall of the four most recently presented items. When items were presented at a slow pace, a substantial increase in RT in a concurrent task was found at items in later positions in the list (Experiments 1 and 2). The RT cost of active updating peaked 1,000 ms following the onset of these later items, but only when they were presented at rates slower than one item per second (Experiment 2). In this condition participants reported employing predominantly active strategies whereas a passive listening strategy was most frequently used at the fast rate. The magnitude of the 1,000 ms peak was larger in running span than in simple span and modified span in Experiment 1, both of which required serial recall only. The active updating of a recall set in running span therefore appears to recruit resources above and beyond those required to encode, store, and rehearse the items in standard serial recall tasks.

Active updating during running span appears to take a relatively fixed period. For the paradigm and stimuli employed in these studies, the peak in cognitive demand occurred 1,000 ms after stimulus presentation. For most participants this limits the reliable application of an active strategy to rates slower than one item per second, as reflected in the strategic shift from active processing to passive listening in the self-report strategy data. Similar time constraints have been noted in other tasks requiring updating. Using a retro-cue paradigm, Oberauer (2001, 2018) found that competition from irrelevant items at retrieval diminished only when the interval between an update cue and a memory probe was longer than 1 s. This time course was not found in the modified span task in which the entire encoded sequence was to be abandoned following a reset cue and encoding of a new sequence started afresh, in line with previous studies with similar tasks (e.g., Kessler & Meiran, 2008).

There are a number of ways in which existing models may be modified to accommodate these findings. One possibility based from the removal account of updating (Kessler & Oberauer, 2014; Lewis-Peacock et al., 2018; Oberauer, 2018) is that the item at the first position is removed and then that the subsequent *n* items are unbound and rebound with the updated ordinal position. The current experimental method may lack the fine-grained temporal resolution necessary to detect successive, rapid changes in item-position bindings, which may indeed be possible only at the level of individual participant data.

Alternatively, the update process might consist of a single binding change in line with the proposed computational model of *n*-back (Chatham et al., 2011). This model imposes a periodicity in serial order such that coding of position *n* + 1, 2*n* + 1 . . . is the same as Position 1. In this way, every new item gets bound to the position associated with the now-irrelevant (first) item of the retrieval set. It could be that the updating costs found here partly reflect the change in the representation of order from linear to periodic although, as yet, it is unclear how this change might occur. A further way of capturing running span without requiring multiple recodings of order is provided by the primacy model of serial recall (Page & Norris, 1998). This represents order in terms of activation strength of items, with the strength decreasing across successive positions. Active updating in this model could be achieved relatively simply by suppressing the first stored item at the beginning of each updating episode. This would reset the activation gradient, allowing the relative order of the already-encoded items to be maintained. Here, the additional cognitive demands would be imposed by the process of suppression.

Finally, we note the substantial self-reported individual differences in the use of strategies in Experiment 2. Active strategies include rehearsal, keeping up with the target set, chunking the sequences, using mental imagery, and semantic recoding into meaningfully related items or sequences (for more, see Morrison, Rosenbaum, Fair, & Chein, 2016). Many participants reported using multiple strategies. Even at the slowest rate of presentation, the keeping-track strategy corresponding to continuous forward-going updating of the target set was reported as being almost always used by only seven of the 30 participants. The same heterogeneity in reported strategies is found in other complex WM tasks including backward span (Norris et al., 2019) and complex span (Minear et al., 2016). The implication is that unfamiliar and cognitively demanding WM tasks are not necessarily served by invariant mechanisms reflected, for example, in a canonical model of running span, but instead reflect flexible and possibly idiosyncratic cognitive solutions (Gathercole et al., 2019). Further experimental investigations of how strategies map onto the more specific cognitive and temporal properties of complex WM tasks will be critical to the resolution of this important theoretical conflict.

## Supplementary Material

10.1037/xlm0000800.supp

## Figures and Tables

**Table 1 tbl1:** Participant Characteristics and Mean ± SDs for Performance in Choice Reaction Time (CRT) Task and Memory Task for Each Load and Task Condition in Experiment 1

Measure	Running span	Modified span	Simple span
Age (*M*, *SD*; years)	25.47, 4.04	23.33, 3.77	24.33, 4.16
Gender	23 f, 7 m	22 f, 8 m	23 f, 7 m
Digit span	7.03 ± 1.33	7.17 ± 1.84	7.30 ± 1.56
CRT (ms)			
Single	425 ± 49	427 ± 55	424 ± 43
Dual	489 ± 90	480 ± 69	447 ± 51
Recall accuracy^a^			
Single	.83 ± .10	.70 ± .21	.77 ± .15
Dual	.76 ± .13	.60 ± .24	.67 ± .16
*Note*. f = female; m = male.			
^a^ Recall scored as proportion of items recalled in correct serial position.			

**Table 2 tbl2:** Mean ± SDs for Performance in Concurrent Choice Reaction Time Task and Running Span Task for Each Load and Rate Condition in Experiment 2

Measure	Fast rate	Medium rate	Slow rate
RT (ms)			
Single	399 ± 43	404 ± 51	403 ± 53
Dual	384 ± 39	401 ± 45	418 ± 57
Recall accuracy^a^			
Single	.79 ± .11	.80 ± .12	.81 ± .11
Dual	.77 ± .13	.77 ± .14	.73 ± .13
*Note.* RT = reaction time.			
^a^ Recall scored as proportion of items recalled in correct serial position.			

## References

[c1] Barrouillet P., Bernardin S., & Camos V. (2004). Time constraints and resource sharing in adults’ working memory spans. Journal of Experimental Psychology: General, 133, 83–100. 10.1037/0096-3445.133.1.83 14979753

[c2] Barrouillet P., Gavens N., Vergauwe E., Gaillard V., & Camos V. (2009). Working memory span development: A time-based resource-sharing model account. Developmental Psychology, 45, 477–490. 10.1037/a0014615 19271832

[c3] Botto M., Basso D., Ferrari M., & Palladino P. (2014). When working memory updating requires updating: Analysis of serial position in a running memory task. Acta Psychologica, 148, 123–129. 10.1016/j.actpsy.2014.01.012 24525166

[c4] Brainard D. H. (1997). The Psychophysics Toolbox. Spatial Vision, 10, 433–436. 10.1163/156856897X00357 9176952

[c5] Broadway J. M., & Engle R. W. (2010). Validating running memory span: Measurement of working memory capacity and links with fluid intelligence. Behavior Research Methods, 42, 563–570. 10.3758/BRM.42.2.563 20479188

[c6] Bunting M., Cowan N., & Saults J. S. (2006). How does running memory span work? The Quarterly Journal of Experimental Psychology, 59, 1691–1700. 10.1080/17470210600848402 16945854 PMC1559727

[c7] Burgess N., & Hitch G. J. (1992). Toward a network model of the articulatory loop. Journal of Memory and Language, 31, 429–460. 10.1016/0749-596X(92)90022-P

[c8] Burgess N., & Hitch G. J. (1999). Memory for serial order: A network model of the phonological loop and its timing. Psychological Review, 106, 551–581. 10.1037/0033-295X.106.3.551

[c9] Chatham C. H., Herd S. A., Brant A. M., Hazy T. E., Miyake A., O’Reilly R., & Friedman N. P. (2011). From an executive network to executive control: A computational model of the n-back task. Journal of Cognitive Neuroscience, 23, 3598–3619. 10.1162/jocn_a_00047 21563882 PMC3269304

[c10] Collette F., Van der Linden M., Laureys S., Arigoni F., Delfiore G., Degueldre C., . . . Salmon E. (2007). Mapping the updating process: Common and specific brain activations across different versions of the running span task. Cortex: A Journal Devoted to the Study of the Nervous System and Behavior, 43, 146–158. 10.1016/S0010-9452(08)70452-0 17334214

[c11] Cowan N. (2001). The magical number 4 in short-term memory: A reconsideration of mental storage capacity. Behavioral and Brain Sciences, 24, 87–114. 10.1017/S0140525X01003922 11515286

[c12] Cowan N., Elliott E. M., Scott Saults J., Morey C. C., Mattox S., Hismjatullina A., & Conway A. R. (2005). On the capacity of attention: Its estimation and its role in working memory and cognitive aptitudes. Cognitive Psychology, 51, 42–100. 10.1016/j.cogpsych.2004.12.001 16039935 PMC2673732

[c13] Craik F. I. M., Govoni R., Naveh-Benjamin M., & Anderson N. D. (1996). The effects of divided attention on encoding and retrieval processes in human memory. Journal of Experimental Psychology: General, 125, 159–180. 10.1037/0096-3445.125.2.159 8683192

[c14] Crowder R. G., & Morton J. (1969). Precategorical acoustic storage (PAS). Perception & Psychophysics, 5, 365–373. 10.3758/BF03210660

[c15] Ecker U. K. H., Lewandowsky S., & Oberauer K. (2014). Removal of information from working memory: A specific updating process. Journal of Memory and Language, 74, 77–90. 10.1016/j.jml.2013.09.003

[c16] Ecker U. K. H., Oberauer K., & Lewandowsky S. (2014). Working memory updating involves item-specific removal. Journal of Memory and Language, 74, 1–15. 10.1016/j.jml.2014.03.006

[c17] Elosúa M. R., & Ruiz R. M. (2008). Absence of hardly pursued updating in a running memory task. Psychological Research, 72, 451–460. 10.1007/s00426-007-0124-4 17851684

[c19] Gathercole S. E., Dunning D. L., Holmes J., & Norris D. (2019). Working memory training involves learning new skills. Journal of Memory and Language, 105, 19–42. 10.1016/j.jml.2018.10.003 PMC6591133 31235992

[c20] Hamilton P., & Hockey R. (1974). Active selection of items to be remembered: The role of timing. Cognitive Psychology, 6, 61–83. 10.1016/0010-0285(74)90004-8

[c21] Hasher L., & Zacks R. T. (1988). Working memory, comprehension, and aging: A review and a new view. Psychology of Learning and Motivation, 22, 193–225. 10.1016/S0079-7421(08)60041-9

[c22] Hasher L., Zacks R. T., & May C. P. (1999). Inhibitory control, circadian arousal, and age In Gopher D. & Koriat A. (Eds.), Attention and performance XVII (pp. 653–675). Cambridge, MA: The MIT Press.

[c23] Hockey R. (1973). Rate of presentation in running memory and direct manipulation of input-processing strategies. The Quarterly Journal of Experimental Psychology, 25, 104–111. 10.1080/14640747308400328

[c24] Jahanshahi M., Saleem T., Ho A. K., Fuller R., & Dirnberger G. (2008). A preliminary investigation of the running digit span as a test of working memory. Behavioural Neurology, 20, 17–25. 10.1155/2008/195684 19491471 PMC5452479

[c25] Jonides J., Smith E. E., Marshuetz C., Koeppe R. A., & Reuter-Lorenz P. A. (1998). Inhibition in verbal working memory revealed by brain activation. Proceedings of the National Academy of Sciences of the United States of America, 95, 8410–8413. 10.1073/pnas.95.14.8410 9653200 PMC20989

[c26] Juvina I., & Taatgen N. A. (2007). Modeling control strategies in the N-back task In Lewis R. L., Polk T. A., & Laird J. E. (Eds.), Proceedings of the Eighth International Conference on Cognitive Modeling (pp. 73–78). Ann Arbor, MI Retrieved from http://act-r.psy.cmu.edu/wordpress/wp-content/uploads/2012/12/718Juvina.pdf

[c27] Kessler Y., & Meiran N. (2008). Two dissociable updating processes in working memory. Journal of Experimental Psychology: Learning, Memory, and Cognition, 34, 1339–1348. 10.1037/a0013078 18980398

[c28] Kessler Y., & Oberauer K. (2014). Working memory updating latency reflects the cost of switching between maintenance and updating modes of operation. Journal of Experimental Psychology: Learning, Memory, and Cognition, 40, 738–754. 10.1037/a0035545 24446752

[c29] Kessler Y., & Oberauer K. (2015). Forward scanning in verbal working memory updating. Psychonomic Bulletin & Review, 22, 1770–1776. 10.3758/s13423-015-0853-0 25962687

[c30] Kiss I., Pisio C., Francois A., & Schopflocher D. (1998). Central executive function in working memory: Event-related brain potential studies. Cognitive Brain Research, 6, 235–247. 10.1016/S0926-6410(97)00035-9 9593912

[c31] Kleiner M., Brainard D., Pelli D., Ingling A., Murray R., & Broussard C. (2007). What’s new in Psychtoolbox-3. Perception, 36, 1–16.

[c32] Lewis-Peacock J. A., Kessler Y., & Oberauer K. (2018). The removal of information from working memory. Annals of the New York Academy of Sciences, 1424, 33–44. 10.1111/nyas.13714 29741212

[c33] McElree B. (2001). Working memory and focal attention. Journal of Experimental Psychology: Learning, Memory, and Cognition, 27, 817–835. 10.1037/0278-7393.27.3.817 PMC3077110 11394682

[c34] Minear M., Brasher F., Guerrero C. B., Brasher M., Moore A., & Sukeena J. (2016). A simultaneous examination of two forms of working memory training: Evidence for near transfer only. Memory & Cognition, 44, 1014–1037. 10.3758/s13421-016-0616-9 27129921

[c35] Morris N., & Jones D. M. (1990). Memory updating in working memory: The role of the central executive. British Journal of Psychology, 81, 111–121. 10.1111/j.2044-8295.1990.tb02349.x

[c36] Morrison A. B., Rosenbaum G. M., Fair D., & Chein J. M. (2016). Variation in strategy use across measures of verbal working memory. Memory & Cognition, 44, 922–936. 10.3758/s13421-016-0608-9 27038310 PMC4976054

[c37] Morton J., Marcus S., & Frankish C. (1976). Perceptual centers (P-centers). Psychological Review, 83, 405–408. 10.1037/0033-295X.83.5.405

[c38] Norris D., Hall J., & Gathercole S. E. (2019). How do we perform backward serial recall? Memory & Cognition, 47, 519–543. 10.3758/s13421-018-0889-2 30771149 PMC6450858

[c39] Oberauer K. (2001). Removing irrelevant information from working memory: A cognitive aging study with the modified Sternberg task. Journal of Experimental Psychology: Learning, Memory, and Cognition, 27, 948–957. 10.1037/0278-7393.27.4.948 11486928

[c40] Oberauer K. (2018). Removal of irrelevant information from working memory: Sometimes fast, sometimes slow, and sometimes not at all. Annals of the New York Academy of Sciences, 1424, 239–255. 10.1111/nyas.13603 29532484

[c41] Oberauer K., Lewandowsky S., Farrell S., Jarrold C., & Greaves M. (2012). Modeling working memory: An interference model of complex span. Psychonomic Bulletin & Review, 19, 779–819. 10.3758/s13423-012-0272-4 22715024

[c42] Page M. P. A., & Norris D. (1998). The primacy model: A new model of immediate serial recall. Psychological Review, 105, 761–781. 10.1037/0033-295X.105.4.761-781 9830378

[c43] Pelli D. G. (1997). The VideoToolbox software for visual psychophysics: Transforming numbers into movies. Spatial Vision, 10, 437–442. 10.1163/156856897X00366 9176953

[c44] Pollack I., Johnson L. B., & Knaff P. R. (1959). Running memory span. Journal of Experimental Psychology, 57, 137–146. 10.1037/h0046137 13641585

[c45] Postle B. R. (2003). Context in verbal short-term memory. Memory & Cognition, 31, 1198–1207. 10.3758/BF03195803 15058681

[c46] Postle B. R., Berger J. S., Goldstein J. H., Curtis C. E., & D’Esposito M. (2001). Behavioral and neurophysiological correlates of episodic coding, proactive interference, and list length effects in a running span verbal working memory task. Cognitive, Affective & Behavioral Neuroscience, 1, 10–21. 10.3758/CABN.1.1.10 12467100

[c47] Ruiz R. M., & Elosúa M. R. (2013). Conditions for positive and negative recencies in running memory-span recognition. Acta Psychologica, 144, 213–223. 10.1016/j.actpsy.2013.07.006 23920403

[c48] Salthouse T. A. (2014). Relations between running memory and fluid intelligence. Intelligence, 43, 1–7. 10.1016/j.intell.2013.12.002 24634555 PMC3951980

[c49] Thalmann M., Souza A. S., & Oberauer K. (2019). Revisiting the attentional demands of rehearsal in working-memory tasks. Journal of Memory and Language, 105, 1–18. 10.1016/j.jml.2018.10.005

[c50] Towse J. N., Hitch G. J., & Hutton U. (1998). A reevaluation of working memory capacity in children. Journal of Memory and Language, 39, 195–217. 10.1006/jmla.1998.2574

[c51] Van Zandt T., & Townsend J. T. (2014). Designs for and analyses of response time experiments In Little T. D. (Ed.), The Oxford handbook of quantitative methods in psychology (Vol. 1, pp. 260–285). New York, NY: Oxford University Press.

