# Supplementary material for: The Time Course of Updating in Running Span
Source: J Exp Psychol Learn Mem Cogn. 2019 Dec 19;46(12):2397–409. doi: 10.1037/xlm0000800 (PMC7790168; doi:10.1037/xlm0000800)
Supplement: Supplementary file 1 [file XLM-2019-0643_Kaur.docx]

| **Table S1**  Post-hoc independent sample t-tests of CRTs between each pair of groups completing different memory tasks in Experiment 1, computed for single and dual CRTs separately. | | | | | | | | | | | | | | |
| --- | --- | --- | --- | --- | --- | --- | --- | --- | --- | --- | --- | --- | --- | --- |
|  | Running span  versus  Modified span | | | |  | Running span  versus  Simple span | | | |  | Modified span  versus  Simple span | | | |
|  | Mean  Diff (ms) | *t  (57)* | *p* | *d* |  | Mean  Diff (ms) | *t  (56)* | *p* | *d* |  | Mean  Diff (ms) | *t  (57)* | *p* | *d* |
| Single CRTs | 3 | .19 | .85 | .05 |  | <1 | .04 | .97 | .01 |  | 3 | .24 | .81 | .06 |
| Dual CRTs | 10 | .47 | .64 | .12 |  | 43 | 2.22 | **.03** | .60 |  | 33 | 2.07 | **.04** | .55 |
| Note: The data analyzed here include all CRTs after trimming and outlier correction, across all lists within each task (for dual condition). Bold text denote significant effects at the p < .05 level, bold italicized text indicate significance effects after adjusting for multiple comparisons using the Bonferroni correction method. | | | | | | | | | | | | | | |

| **Table S2**  Post-hoc independent sample t-tests of dual CRTs between each pair of tasks in Experiment 1, analyzed separately for early (1 to 4) and late (5 and 6) positions. | | | | | | | | | | | | | | |
| --- | --- | --- | --- | --- | --- | --- | --- | --- | --- | --- | --- | --- | --- | --- |
|  | Running span  versus  Modified span | | | |  | Running span  versus  Simple span | | | |  | Modified span  versus  Simple span | | | |
|  | Mean  Diff (ms) | *t  (57)* | *p* | *d* |  | Mean  Diff (ms) | *t  (56)* | *p* | *d* |  | Mean  Diff (ms) | *t  (57)* | *p* | *d* |
| Early positions | 3 | .20 | .84 | .05 |  | 22 | 1.40 | .17 | .37 |  | 19 | 1.30 | .20 | .34 |
| Late positions | 61 | 1.89 | .06 | .52 |  | 80 | 2.57 | ***.01*** | .74 |  | 19 | 1.04 | .30 | .27 |
| Note: The data analyzed here include dual CRTs after trimming and outlier correction, across the first six positions as these were comparable across the three tasks. Bold text denote significant effects at the p < .05 level, bold italicized text indicate significance effects after adjusting for multiple comparisons using the Bonferroni method. | | | | | | | | | | | | | | |

| **Table S3**  Results from the omnibus 6x2x3 ANOVA to investigate the effect of bin, position, and task in Experiment 1. | | | | | |
| --- | --- | --- | --- | --- | --- |
|  | *F* | *df* | *p* | $\eta_{p}^{2}$ |  |
| Bin | 8.81 | 3.25,6.50 | **< .001** | .09 |  |
| Position | 64.54 | 1,85 | **< .001** | .43 |  |
| Task | 2.96 | 2,85 | .057 | .07 |  |
| Bin * Position | 27.30 | 3.63,308.24 | **< .001** | .24 |  |
| Bin * Task | 2.73 | 6.50,276.41 | **.01** | .06 |  |
| Position * Task | 6.60 | 2,85 | **.002** | .13 |  |
| Bin * Position * Task | 2.56 | 7.25,308.24 | **.01** | .06 |  |
| *Note:* Bin was a within-subject factor with six levels (the 2400 ms interval between consecutive items was divided into six 400 ms bins). Position was a within-subject factor with two levels (early positions 1 to 4 versus late positions 5 and 6). Task was a between-group factor with three levels (running span, simple span, and modified span). The data analyzed here include dual CRTs after trimming and outlier correction extracted from the first six positions as those were comparable across tasks. Bold text denote significant effects at the *p* < .05 level. | | | | | |

| **Table S4**  Post-hoc 2x3 ANOVAs to investigate the interaction between bin and task for each pair of consecutive bins across the three memory tasks, and difference between means where applicable. | | | | | | | | |
| --- | --- | --- | --- | --- | --- | --- | --- | --- |
|  | Task * Bin interaction effect | | | |  | Mean difference between bins (ms) | | |
|  | *F* | *df* | *p* | $\eta_{p}^{2}$ |  | Running | Modified | Simple |
| Bin 1 vs 2 | 1.46 | 2,85 | .24 | .03 |  | . | . | . |
| Bin 2 vs 3 | 3.76 | 2,85 | **.03** | .08 |  | ***40*** | ***18*** | ***12*** |
| Bin 3 vs 4 | 3.95 | 2,85 | **.02** | .09 |  | - 21 | 6 | **8** |
| Bin 4 vs 5 | 1.84 | 2,85 | .17 | .04 |  | . | . | . |
| Bin 5 vs 6 | 1.41 | 2,85 | .25 | .03 |  | . | . | . |
| *Note:* The data analyzed here include dual CRTs after trimming and outlier correction extracted from late positions (five and six). Pairwise analyses were conducted only if the task * bin interaction for that bin-pair was significant. An increase in RT between consecutive bins is indicated in positive mean difference, while a decrease is indicated in negative values. Bold text denote significant effects at the *p* < .05 level, bold italicized text indicate significance effects after adjusting for multiple comparisons using the Bonferroni method. Only data related to late positions provided here as there was no overall bin*task interaction during in the 6x3 ANOVA conducted for early positions (see text). | | | | | | | | |

| **Table S5**  Post-hoc 2x2 ANOVAs to investigate the interaction between bin and task for each pair of consecutive bins between the two WM tasks involving updating (running span and modified span), and difference between means where applicable. | | | | | | |
| --- | --- | --- | --- | --- | --- | --- |
|  | Task * Bin interaction  in post-hoc 2x2 ANOVAs | | | | Mean difference  between bins (ms) | |
|  | *F* | *df* | *p* | $\eta_{p}^{2}$ | Running | Modified |
| Bin 1 and 2 | .18 | 1,57 | .67 | .003 | . | . |
| Bin 2 and 3 | 25.62 | 1,57 | ***< .001*** | .31 | ***36*** | ***- 27*** |
| Bin 3 and 4 | 2.54 | 1,57 | .12 | .04 | . | . |
| Bin 4 and 5 | .81 | 1,57 | .37 | .01 | . | . |
| Bin 5 and 6 | 1.29 | 1,57 | .26 | .02 | . | . |
| *Note:* The data analyzed here include dual CRTs after trimming and outlier correction extracted from update positions for two memory tasks (running span: positions 5-12; modified span: positions: 8, 15, and 22 in the sequence). Pairwise analyses were conducted only if the task * bin interaction for that bin-pair was significant. An increase in RT between consecutive bins is indicated in positive mean difference, while a decrease is indicated in negative values. Bold text denote significant effects at the *p* < .05 level, bold italicized text indicate significance effects after adjusting for multiple comparisons using the Bonferroni method. | | | | | | |

| **Table S6**  Post-hoc paired-sample t-tests of recall accuracy in single and dual task, performed separately for each target position and memory task. | | | | | | | | | | | |
| --- | --- | --- | --- | --- | --- | --- | --- | --- | --- | --- | --- |
|  | Running span | | |  | Modified span | | |  | Simple span | | |
|  | Recall in  single versus dual task | | |  | Recall in  single versus dual task | | |  | Recall in  single versus dual task | | |
|  | *t* | *p* | *Cohen’s d* |  | *t* | *p* | *Cohen’s d* |  | *t* | *p* | *Cohen’s d* |
| Position 1 | 6.17 | ***< .001*** | 1.16 |  | 3.27 | ***.003*** | .89 |  | 3.87 | ***.001*** | .75 |
| Position 2 | 3.89 | ***.001*** | .76 |  | 3.83 | ***.001*** | .83 |  | 4.01 | ***< .001*** | .76 |
| Position 3 | 2.61 | **. 014** | .52 |  | 7.27 | ***< .001*** | 1.41 |  | 6.04 | ***< .001*** | 1.15 |
| Position 4 | .99 | .330 | .19 |  | 7.54 | ***< .001*** | 1.41 |  | 5.78 | ***< .001*** | 1.07 |
| Position 5 | . | . | . |  | 5.36 | ***< .001*** | .98 |  | 7.04 | ***< .001*** | 1.28 |
| Position 6 | . | . | . |  | 4.45 | ***< .001*** | .88 |  | 7.02 | ***< .001*** | 1.33 |
| Position 7 | . | . | . |  | 2.66 | **. 013** | .44 |  | 2.5 | **.018** | .45 |
| *Note:* Bold text denote significant effects at the *p* < .05 level, bold italicized text indicate significance effects after adjusting for multiple comparisons using the Bonferroni correction method. | | | | | | | | | | | |

| **Table S7**  Paired sample t-tests of CRTs between each pair of rate conditions in Experiment 2 to test the hypothesis that the difference between fast and slow rates would be greater for dual than single CRTs. | | | | | | | | | | | | | | |
| --- | --- | --- | --- | --- | --- | --- | --- | --- | --- | --- | --- | --- | --- | --- |
|  | Fast versus Medium | | | |  | Fast versus Slow | | | |  | Medium versus Slow | | | |
|  | Mean  Diff (ms) | *t  (28)* | *p* | *d* |  | Mean  Diff (ms) | *t  (28)* | *p* | *d* |  | Mean  Diff (ms) | *t  (28)* | *p* | *d* |
| Single CRTs | 5 | .72 | .48 | .13 |  | 4 | .58 | .56 | .11 |  | <1 | .08 | .94 | .01 |
| Dual CRTs | 17 | 2.74 | ***.01*** | .51 |  | 33 | 4.08 | ***< .001*** | .81 |  | 17 | 2.21 | **.04** | .42 |
| Note: The data analyzed here include all CRTs after trimming and outlier correction, across all lists within each rate. Bold text denote significant effects at the *p* < .05 level, bold italicized text indicate significance effects after adjusting for multiple comparisons using the Bonferroni correction method. | | | | | | | | | | | | | | |

| **Table S8**  Paired sample t-tests of CRTs between each pair of rate conditions in Experiment 2 to test the hypothesis that the difference between fast and slow rates would be greater for CRTs during late than early positions. | | | | | | | | | | | | | | |
| --- | --- | --- | --- | --- | --- | --- | --- | --- | --- | --- | --- | --- | --- | --- |
|  | Fast versus Medium | | | |  | Fast versus Slow | | | |  | Medium versus Slow | | | |
|  | Mean  Diff (ms) | *t  (28)* | *p* | *d* |  | Mean  Diff (ms) | *t  (28)* | *p* | *d* |  | Mean  Diff (ms) | *t  (28)* | *p* | *d* |
| Early | 11 | 1.83 | .08 | .35 |  | 22 | 3.03 | ***.005*** | .31 |  | 11 | 1.71 | .10 | .32 |
| Late | 28 | 3.48 | ***.002*** | .31 |  | 48 | 4.0 | ***< .001*** | .31 |  | 20 | 1.78 | **.**09 | .31 |
| Note: The data analyzed here include all dual CRTs after trimming and outlier correction, across all lists within each rate. Early CRTs are those at positions one to four, and late CRTs are from positions five onward. Bold text denote significant effects at the *p* < .05 level, bold italicized text indicate significance effects after adjusting for multiple comparisons using the Bonferroni correction method. | | | | | | | | | | | | | | |

| **Table S9**  2x2 ANOVAs to examine the interaction between bin and position for each pair of consecutive bins between early and late positions within the slow rate condition in Experiment 2, and pairwise mean differences in RT where applicable, to test timing of the hypothesized peak in RT between bins. | | | | | | | |
| --- | --- | --- | --- | --- | --- | --- | --- |
|  | Bin * Position interaction effect | | | |  | Pairwise mean diff in RT | |
|  | *F* | *df* | *p* | $\eta_{p}^{2}$ |  | Early | Late |
| Bin 1 vs 2 | 1.25 | 1,28 | .27 | .04 |  | . | . |
| Bin 2 vs 3 | 17.66 | 1,28 | ***< .001*** | .39 |  | 2 | ***18*** |
| Bin 3 vs 4 | 8.99 | 1,28 | ***.006*** | .24 |  | 2 | ***10*** |
| *Note:* The data analyzed here include dual CRTs in the slow rate condition after trimming and outlier correction extracted from early (one to four) and late positions (five onward). Pairwise analyses were conducted only if the bin * position interaction for that bin-pair was significant. An increase in RT between consecutive bins is indicated in positive *Cohen’s d* values, while a decrease is indicated in negative values. Bold text denote significant effects at the *p* < .05 level, bold italicized text indicate significance effects after adjusting for multiple comparisons using the Bonferroni method. | | | | | | | |

| **Table S10**  Post-hoc paired-sample t-tests of recall accuracy in single and dual task, performed separately for each target position and rate condition in Experiment 2. | | | | | | | | | | | |
| --- | --- | --- | --- | --- | --- | --- | --- | --- | --- | --- | --- |
|  | Fast rate | | |  | Medium rate | | |  | Slow rate | | |
|  | Recall in  single versus dual task | | |  | Recall in  single versus dual task | | |  | Recall in  single versus dual task | | |
|  | *t* | *p* | *Cohen’s d* |  | *t* | *p* | *Cohen’s d* |  | *t* | *p* | *Cohen’s d* |
| Position 1 | 1.46 | .16 | .28 |  | 2.60 | **.02** | .49 |  | 5.61 | ***< .001*** | 1.04 |
| Position 2 | 1.56 | .13 | .29 |  | 2.04 | **.05** | .40 |  | 5.75 | ***< .001*** | 1.13 |
| Position 3 | 1.74 | .09 | .36 |  | 1.55 | .13 | .29 |  | 2.97 | ***.006*** | .62 |
| Position 4 | .27 | .78 | .05 |  | 1.68 | .10 | .31 |  | 3.18 | ***.004*** | .59 |
| *Note:* Bold text denote significant effects at the *p* < .05 level, bold italicized text indicate significance effects after adjusting for multiple comparisons using the Bonferroni correction method. | | | | | | | | | | | |
